# Supplementary material for: Construction and validation of the area level deprivation index for health research: A methodological study based on Nepal Demographic and Health Survey
Source: PLoS One. 2023 Nov 16;18(11):e0293515. doi: 10.1371/journal.pone.0293515 (PMC10653511; doi:10.1371/journal.pone.0293515)
Supplement: S1 File — (PDF) [file pone.0293515.s001.pdf]

APPENDIX

Table S1: List of variables included for the construction of area level deprivation

	Variables for the area level deprivation
**1**	% of the disadvantageous/marginalized population
**2**	% of the dependent population
**3**	% of households with illiterate male/female
**4**	% of households with television
**5**	% of households with refrigerator
**6**	% of households with motorbike
**7**	% of households with cycle
**8**	% of households with car
**9**	% of households with radio
**10**	% of households with separate kitchen as cooking rooms
**11**	% of households with electricity
**12**	% of households with toilet
**13**	% of households with clean drinking water
**14**	% of households with shared toilet
**15**	% of households with rudimentary floor
**16**	% of households with rudimentary wall
**17**	% of households with phone
**18**	% of households with clean energy source
**19**	% of households with bank account
**20**	% of households with employed members
**21**	% of households exposed to mass media
**22**	% of household with soap
**23**	Average time to reach the nearest health facility (time in minutes)
**24**	Average time to reach the nearby motorable road (time in minutes)
**25**	Average time to reach to the nearby water source (time in minutes)
**26**	Altitude (in meters)

Table S2: Pearson correlation coefficients for the 15 variables selected for the Area level Deprivation index construction and their mean and standard deviations (last line)

	Edu	Occ	TV	Ref	Mcyc	Flr	Wall	T_Ph	Ene	Soap	Bacc	Mmed	T_Wt	T_Rd	Elec
**Edu**	1														
**Occ**	0.52	1													
**TV**	0.52	0.52	1												
**Ref**	0.64	0.57	0.60	1											
**Mcyc**	0.45	0.53	0.62	0.71	1										
**Flr**	0.67	0.65	0.70	0.82	0.70	1									
**Wall**	0.51	0.55	0.64	0.70	0.67	0.84	1								
**T_Ph**	0.47	0.42	0.42	0.70	0.56	0.59	0.48	1							
**Ene**	0.71	0.63	0.64	0.86	0.68	0.86	0.78	0.62	1						
**Soap**	0.57	0.52	0.51	0.59	0.50	0.61	0.56	0.42	0.63	1					
**Bacc**	0.48	0.41	0.55	0.46	0.42	0.53	0.50	0.31	0.48	0.44	1				
**Mmed**	0.52	0.46	0.79	0.51	0.43	0.59	0.58	0.34	0.58	0.51	0.54	1			
**T_Wt**	0.28	0.28	0.40	0.35	0.41	0.39	0.43	0.24	0.36	0.41	0.29	0.35	1		
**T_Rd**	0.29	0.34	0.57	0.36	0.46	0.46	0.52	0.15	0.40	0.41	0.46	0.55	0.45	1	
**Elect**	0.26	0.39	0.48	0.27	0.29	0.33	0.36	0.18	0.32	0.34	0.42	0.51	0.35	0.22	1
Mean	*0.38*	*0.47*	*0.48*	*0.84*	*0.81*	*0.64*	*0.53*	*0.93*	*0.71*	*0.52*	*0.29*	*0.31*	*0.30*	*84.3*	*0.10*
(SD)	*0.16*	*0.22*	*0.32*	*0.22*	*0.19*	*0.35*	*0.35*	*0.13*	*0.35*	*0.30*	*0.22*	*0.28*	*0.30*	*117.5*	*0.20*

Edu: Proportion of illiterate respondents, Occ: proportion of households without employment/unskilled/agriculture , TV: Proportion of households without television; Ref: Proportion of households without refrigerator, Mcyc: Proportion of households with motorcycle, Flr: Proportion of households with rudimentary floor; Wall: proportion of households with rudimentary wall; T-Ph: proportion of households with telephone; Ene: Proportion of households with energy source; Soap: Proportion of households with soap in washroom; Bacc: Proportion of households with bank account; Mmed: Proportion of households without exposure to mass media; T_wt: Average time required to collect drinking water; T_Rd: Average time required to reach nearby motorable roads, Elec: Proportion of households with electricity.

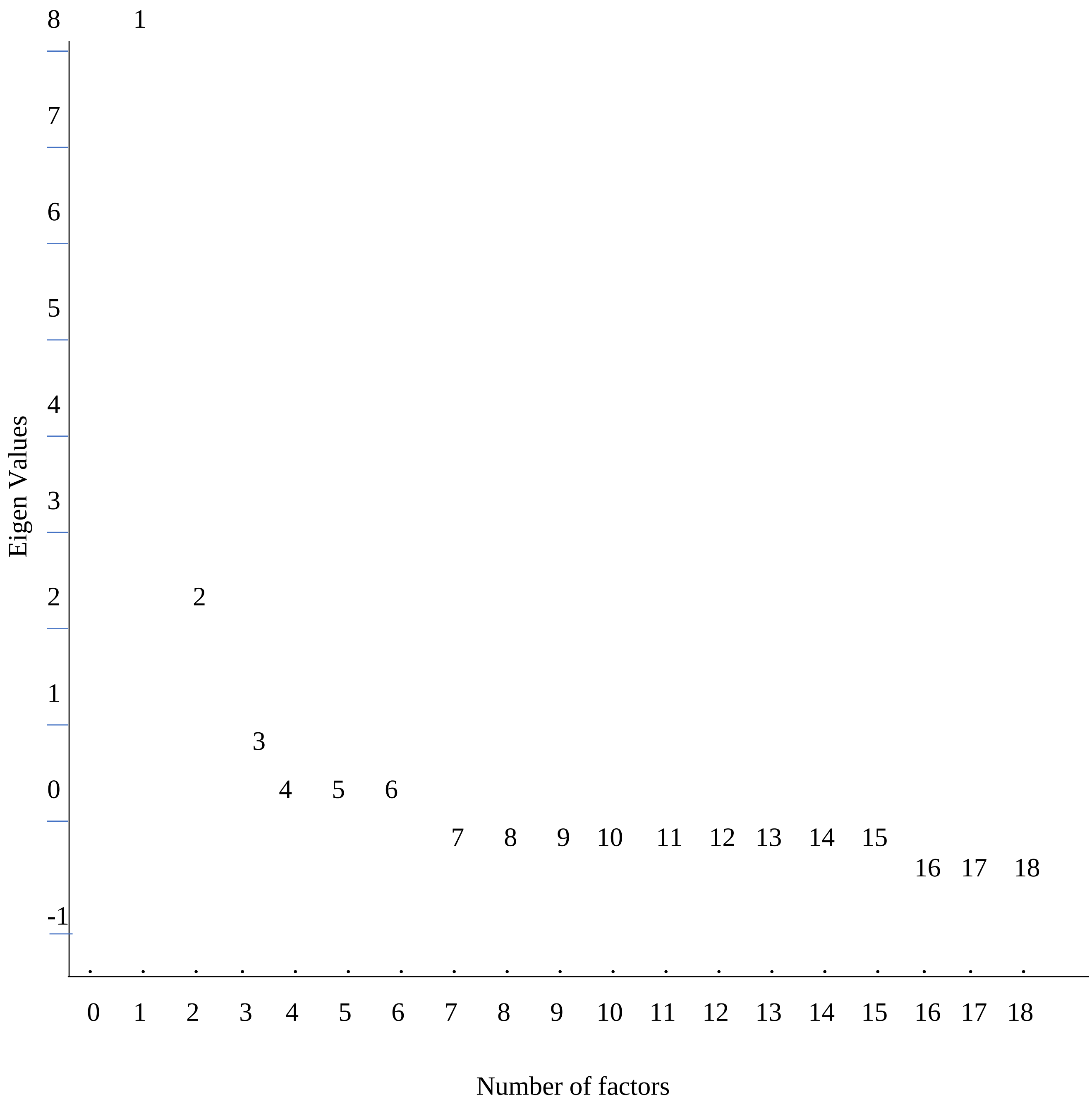

S1 Figure: Scree plot of eigen values vs. number of factors

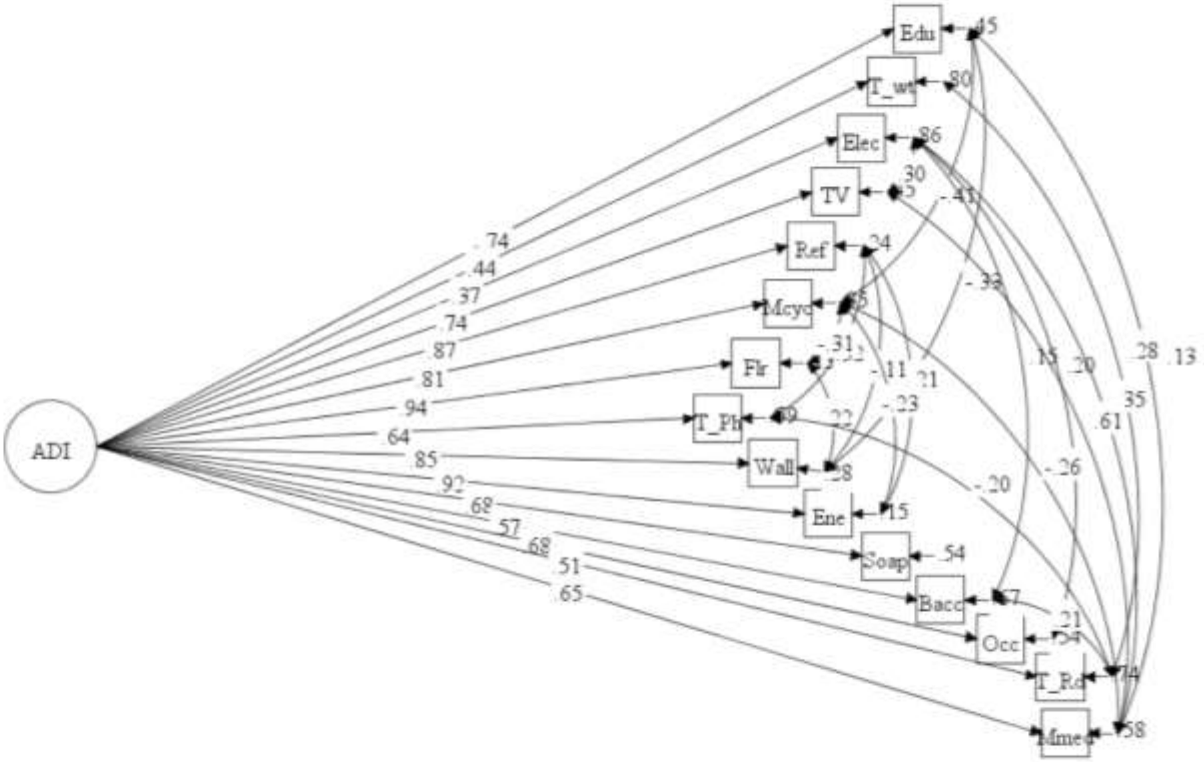

S2 Figure: Unidimensional model of 15-item Area Level Deprivation Index (ADI) for Nepal based on Nepal Demographic Health survey-2016. Rectangles representing observed/manifest variables; Circle represent the latent variable; single headed arrows from latent variable to observed variable represent the factor loadings. Single headed errors towards the observed variables shows residual error. Curved lines show the error term correlation. Model fit: Comparative Fit Index = 0.96; Tucker-Lewis Index= 0.94; standardized root mean square residual = 0.05; Root mean square error of approximation = 0.079
